# Teaching bioscience to nursing students—What works?

**DOI:** 10.1002/nop2.709

**Published:** 2020-11-25

**Authors:** Unni Knutstad, Milada Cvancarova Småstuen, Kari Toverud Jensen

**Affiliations:** ^1^ Oslo Metropolitan University Oslo Norway

**Keywords:** bioscience, education, flipped classroom, nurses, nursing, students

## Abstract

**Aim:**

To compare the effects of flipped classroom and traditional auditorium lectures, on nursing students’ examination results in bioscience.

**Design:**

An educational intervention study.

**Methods:**

All the first‐year students in the bachelor programme (*N* = 493) were entered into a database and randomly assigned to the intervention or the control group in a course in bioscience. The outcome measures are the proportion of students who passed the examination, and the distribution of grades from A to E. Chi‐square tests and Mann–Whitney Wilcoxon test were used. The odds to pass versus fail were modelled using binary logistic regression.

**Results:**

The proportion of students who did not pass the final examination was very similar in the intervention and the control groups, 21.4% and 23.6% (*p* = .574). Our data did not reveal any statistically significant differences concerning the distribution of grades (*p* = .691). Students with biology and/or natural science had higher odds for passing.

## 1 INTRODUCTION

Bioscience is a key area of nursing education and is often understood as a prerequisite for nursing practice and for understanding the discipline of nursing. The overall goal of this project is to develop and improve the bioscience courses by testing alternative teaching methods against student performance.

## 2 BACKGROUND

The learning of bioscience in nurse education has received much attention in recent years due to the nursing students’ struggle to acquire the bioscience knowledge and later apply the knowledge to clinical practice (Andrew et al., 2015; Bakon et al., 2016; Johnston et al., 2015; McVicar et al., 2015; Molesworth & Lewitt, 2016; Salamonson et al., 2016; Salvage‐Jones et al., 2016). Furthermore, Sulosaari et al. (2011) point to knowledge of anatomy and physiology as a primary foundation for understanding the principles of pharmacology. The biosciences of nurse education include anatomy, physiology and biology. Most of the bioscience courses are placed in the first and second year and are thought of as more or less a separate discipline (Jensen et al., 2018). In addition, the bioscience courses in countries such as Norway are generally taught by physiologists or medically trained personnel, which contribute to separate the biosciences from the rest of the nursing curriculum. Salamonson et al. (2016), using a group of 563 first‐year nursing students, examined the relationship between the sense of coherence, self‐regulated learning and academic performance in the subject of bioscience. The findings indicated that students with a higher sense of coherence were better able to adopt self‐regulated learning strategies and achieve higher academic grades. According to Gordon et al. (2017), young students with no background in science from secondary school judged bioscience as hard to learn. Rooyen et al. (2006) found that the nursing school entry criteria in bioscience showed a strong relationship to the students’ performance in bioscience the first year of study.

Jensen et al. (2018) argue that the research field addressing the teaching of bioscience in nursing could benefit from a broader range of inquiry to provide a better understanding of this issue, which Fawcett et al. (2016) have described, as a forgotten priority in nursing. There seems to be a lack of knowledge related to different teaching methods and their effect on students’ learning outcomes in bioscience (Jensen et al., 2018; McVicar et al., 2015). However, Salvage‐Jones et al. (2016) performed a study of a teaching method among undergraduate nurse students in anatomy and physiology. The method consisted of practical bioscience activities, such as games, puzzles and laboratory activities. Despite positive responses, the students’ test performances did not improve.

One intervention of interest in nurse education is the use of “flipped classroom,” the chosen intervention in this study. Flipped classroom is a pedagogical approach characterized by shifting the learning space from the traditional auditorium to individual preparation and problem‐solving in groups. The content, which in traditional lectures is presented in a classroom setting, is, in a flipped classroom, assigned before class as homework, and the time in class is dedicated to active learning (Herreid & Schiller, 2013). A literature review of the use of flipped classrooms in nurse education describes mainly the implementation of the intervention and to a lesser extent how the intervention relates to (or is effective on) students’ achievements (Presti, 2016). Chen et al. (2017) did a systematic literature review on the effectiveness of the flipped classroom teaching approach in medical education. They concluded that "flipped classroom" is a promising teaching method, especially considering the students’ motivation, the value of and commitment to the task and found that the method was at least as effective as traditional teaching, with regard to the students' knowledge and skills. Harrington et al. (2015) studied nursing students in flipped classroom and found no significant differences in outcome measures; examination questions, quiz scores and course grades, between flipped or the traditional classroom. Missildine et al. (2013) performed a quasi‐experimental study of different teaching approaches in adult health nursing, including the flipped classroom. Their results show that students’ examination scores were higher in the flipped classroom, but the students were less satisfied with the method compared with the traditional methods.

To the best of our knowledge, there is a gap in the literature regarding the impact of the flipped classroom teaching approach on students’ performance in bioscience. Differences in the research results pave the way for a new study of the flipped classroom approach and its effect on student outcomes.

## 3 THE AIM

The aim of this study is to compare the effects of two teaching methods, flipped classroom and traditional auditorium lectures, on nurse students’ examination results in bioscience:

H0: There is no difference between flipped classroom and traditional lectures regarding the proportion of students who pass their bioscience examination.
H1: Flipped classroom has a positive effect on the proportion of students who pass their bioscience examination, when compared with traditional classroom lectures.

## 4 THE STUDY

The course in bioscience was offered from August–December 2018, and the course was completed by a final, four hours examination.

## 5 DESIGN

The study was carried out as a complex intervention study. The intervention study is described as a complex intervention, based on the Medical Research Council's (MRC) key elements on how to develop complex interventions (Craig et al., 2019, p. 8).

What makes an intervention complex is described by Craig and Petticrew (2013) as

- Number of interacting components within the experimental and control interventions.
- Number and difficulties of behaviours required by those delivering or receiving the intervention.
- Number of groups or organizational levels targeted by the intervention.
- Number and variability of outcomes.
- Degree of flexibility or tailoring of the intervention permitted (s. 588).

The objective of choosing a complex intervention design for educational research is to distinguish the effect on student grades of the different components of student learning, such as different teaching methods, the cooperation between students, how much the students read and the learning context. Four different teachers participated in the intervention in this study. Flipped classroom was a new method for all four teachers, which may have an impact on our results. One advantage may occur, that the teachers were motivated to learn a new method and play by new rules, one drawback could be the lack of experience with the method.

## 6 METHODS

All the first‐year students in the bachelor programme at Oslo Metropolitan University (*N* = 493) were entered into the National Students database and randomly assigned to two classes. This is the normal procedure for dividing students into different classes. One of the classes became the intervention group and the other the control group. This study was conducted as a randomized controlled trial (RCT). The participants were randomized to minimize the effect of possible baseline differences and confounding. The intervention took place during August through November 2018 in a first‐level theory course in anatomy, physiology and biology. As of 2015, a national assessment examination has been given in biosciences in bachelor programmes in nursing.

The main outcome measure is the proportion of students who passed the bioscience examination for nursing students. The secondary outcome is distribution of grades from A–E.

### 6.1 Development of the intervention

First, a literature review was conducted to explore the literature. This study showed that there were few intervention studies and thereby knowledge of how best to effectively support students’ learning. Students appear satisfied with the bioscience courses but there appears to be no correlation between satisfaction and achievement (Jensen et al., 2018, p. 1794). The theoretical background of this study is learning in the professions (Benner et al., 2010; Sullivan, 2005).

### 6.2 Piloting

Secondly, the intervention in bioscience was piloted in a small group in a radiography programme at the same university. The students achieved higher results than the control group (unpublished). The design of this study was based on an educational bioscience intervention developed and implemented in the radiography bachelor programmes. The responsible teacher from the radiography programme was mentor for the intervention and based the intervention on experience from the radiography programme.

### 6.3 Implementation

The intervention group included 238 nursing students divided into smaller groups. Students were offered four‐hour seminars each week for 11 weeks. The students used a digital platform (Book cabinet) for lectures and assignments. Various learning activities were used to enhance understanding of anatomy, physiology and biochemistry. The flipped classroom approach in this study comprises the students' activities before, during and after the classroom meeting. Students are recommended to study relevant lectures and literature before class, using digital devices, textbooks and articles. During the classroom meetings, the students discuss their reading and help develop each other's understanding of relevant concepts in bioscience. Furthermore, the students take part in different learning activities, such as palpation, drawing and testing each other's knowledge. The teachers are present in class to resolve challenging issues and to ensure that the sessions are carried out according to schedule. After the classroom meetings, students are advised to check their understanding and continue their learning process in student groups.

The control group consisted of 255 students. This group was offered auditorium lectures for all 255 students together, 4 hr of lectures each week for 12 weeks, given by a Medical Doctor. The lectures were open to all students, including those in the intervention group. The topics were largely taught in the same order for both groups, starting with cells and tissues, muscle and skeleton, and ending with the nervous system and reproduction.

### 6.4 Evaluation

MRC guidance for evaluation of complex interventions focuses mainly on randomized trials, whereas process evaluation receives little attention. Moore et al. (2015) underline that process evaluation can be used to assess fidelity and quality of implementation, clarify causal mechanisms and identify contextual factors associated with variation in outcomes, but better guidance for carrying out process evaluation is needed.

### 6.5 Statistical analyses

All analyses were performed according to the intention to treat (ITT) principle. Categorical variables were described as counts with proportions. Age, a continuous variable, was presented as median and range (min and max value). Possible differences between groups concerning background variables were assessed using chi‐square tests (categorical data) and Mann–Whitney Wilcoxon test (continuous data).

In addition, the odds to pass versus fail an examination were modelled using binary logistic regression. Background variables (gender, age and prior education in natural science and biology) were entered as possible confounders, and the results are expressed as odds ratios (OR) with 95% confidence intervals (CI). All tests were two‐sided, and *p*‐values <.05 were considered statistically significant. All analyses were performed using SPSS version 24.

## 7 RESULTS

In total, we collected data on 493 students randomized into two groups, 238 in the intervention group and 255 in the control group. Background characteristics (gender and age distribution and prior education in natural science and biology) were similar in both groups (Table 1). As can be seen from Table 1, there were no baseline differences between the groups, that is we managed to achieve a balanced design and no correction was thus necessary for possible confounding. Moreover, a similar proportion of students in both groups (13.5% in intervention group and 13.7% in control group) did not participate in the final examination (did not attend, were sick or dropped out) (Table 2.).

**Table 1:** Background characteristics

	Intervention group		Control group		*p*‐value^a^
	*N*	%	*N*	%	
Gender					
Female	197	82.8	213	83.5	.823
Male	41	17.2	42	16.5	

	Median	Range (min. maks)	Median	Range (min. maks)	
Age	21	19–45	21	19–53	.442

	*N*	%	*N*	%	
Science level from high school					
1–3	23	9.7	32	12.5	.673
≤4	185	77.7	193	75.7	
Does not have Norwegian education	30	12.6	30	11.8	
Biology (high school, level 1)					
Has bio	35	14.7	47	18.4	.267
Does not have prior bio	203	85.3	208	81.6	
Biology 2 (high school level 2)					
Has bio 2	19	8.0	24	9.4	.574
Does not have prior bio	219	92.0	231	90.6	

^a^ All *p*‐values refer to chi‐square tests except of *p*‐value for age. Age was compared using Mann–Whitney Wilcoxon test.

**Table 2:** Students pass/no pass the bioscience examination in nurse education

Examination results	Intervention group		Control group		*p*‐value
	*N* = 238	%	*N* = 255	%	
Pass	162	68.1	168	65.9	.574 (passed vs. not passed)
No pass	44	18.5	52	20.4	
Sick	8	3.4	12	4.7	
Not attended	5	2.1	1	0.4	
Dropped out	19	8.0	22	8.6	

We have analysed the results of 206 students in the intervention group and 220 in the control group. Concerning the main outcome, the proportion of students who did not pass the final examination was very similar in the intervention and the control groups, 21.4% and 23.6%, in the intervention and the control group, respectively (*p* = .574).

The distribution of achieved grades can be seen in Table 3. Our data did not reveal any statistically significant differences concerning the distribution of grades (*p* = .691).

**Table 3:** Distribution of grades of the bioscience examination in nurse education

Examination grade	Intervention group		Control group		*p*‐value
	*N* = 206		*N* = 220		.691
A	30	14.6	34	15.5	
B	35	17.0	44	20.0	
C	54	26.2	51	23.2	
D	32	15.5	34	15.5	
E	11	5.3	5	2.3	
F (not passed)	44	21.4	52	23.6	

To control for possible confounding factors like previous education in biology or natural science, and age and gender, we have fitted a multiple logistic regression model. Our results revealed that none of the included variables were significantly associated with higher odds to pass an examination.

Students who had studied biology and/or natural science had higher odds for passing the examination; however, the results did not reach the level of statistical significance, see Table 4.

**Table 4:** Odds for passing an examination (only students who took the test are included). Logistic regression analysis

	OR	95% CI	*p*‐value
Group Ref = grade A	1.13	0.72–1.80	.584
Age	1.02	0.97–1.08	.357
Science 1(S1) background Ref = does not have S1	1.80	0.91–3.57	.093
Science 2 (S2) Ref = does not have S2	1.545	0.57–4.17	.391
Biology Ref = does not have	1.73	0.86–3.49	.127

Prior education in natural science showed a trend for passing for those who had studied natural science before (*p* = .09). Students with such education were 1.8 times more likely to pass an examination compared with those without any background in natural science.

In this study, the hypothesis 0 “There is no difference between flipped classroom and traditional lectures regarding the proportion of students who pass their bioscience exam” is therefore not rejected.

## 8 DISCUSSION

In this study, the intervention “flipped classroom” showed no effect on students’ probability to pass or not pass, nor on the distribution of the examination grades (A‐E), compared with the traditional lectures. The hypothesis for this study (H1) that the flipped classroom has a positive effect on the proportion of students who pass their bioscience examination could not be supported by our data, as the results were comparable with those achieved by students randomized to the traditional lectures.

However, our data revealed that students with prior education in natural science were more likely to pass biosciences examinations. Several discussions are currently going on in Norway, both politically and professionally, regarding entry levels and entry grades for higher education. In nurse education, certain grades in languages and mathematics are required from 2019, but not in biology and natural science. Our result may actualize a discussion about the need for certain entry grades in biosciences or natural sciences.

Prior work has documented none or small differences in nursing student outcomes achieved using different teaching methods (Chen et al., 2017; Harrington et al., 2015; Missildine et al., 2013). Our intervention study showed no effect of the flipped classroom on examination grades compared with traditional lectures in biosciences. Based on the results of our study and earlier research, the discussion will concentrate on three topics: limitation of the intervention, the importance of relevance and the bioscience course's position in the curriculum.

### 8.1 The limitations of the intervention

This is a complex intervention in an educational context. Moore et al. (2015) point out two sets of explanations of limited effects: weakness in design or weakness in intervention implementation.

One of the weaknesses in the intervention design is a lack of evaluation of the intervention process. Another weakness is the translation of the intervention from a small programme in radiography to a huge educational programme in nursing. The sizes of the education groups in nursing and radiography are radically different, and the two professions have different tasks in health care. The faculty of radiography developed the examination for that programme, while a national expert group developed the examination in biosciences for nursing students. Any or several of these factors may have limited the effect of the intervention.

There were several interacting components within the experimental and control group. Firstly, attending the bioscience course is not compulsory; the students choose whether to attend the course or to study on their own. The flipped classroom students could choose to attend the traditional lectures; the control group students, however, were not allowed into the intervention group. The possibilities for students to choose learning activities outside the intervention group increase the intervention complexity. Secondly, the teachers who were responsible for the intervention group had no earlier experience with the method, and this may be a factor that influenced the quality of the intervention. The teachers as a group, however, had regular meetings to discuss the teaching method, and they adjusted the intervention en route.

### 8.2 Difficulties of behaviours required by those delivering or receiving the intervention

Among the teachers who delivered the intervention, only one of four supervisors was a RN, and this may have affected the effort of making bioscience relevant to the nurse profession. It is argued that the academic staff needs closer links with practice so that learners are able to immediately identify the content's relevance to the practice placement reality (Hatlevik & Smeby, 2015). According to Jensen et al. (2018), bioscience courses are internationally often seen as a separate discipline from nursing. For a teacher with a background from other professions, it may be demanding to find and use examples that are relevant for nurses, such as the relevance of bioscience for nurses’ observations of patients, for understanding patients’ expression of disease and for understanding the treatment of the patients' basic needs. Physiologists, on the other hand, may provide examples relevant to bioscience, and MDs are usually more focused on the causes, the investigations and treatments of disease.

Differences also exist in the group receiving the intervention (the students). According to Salamonson et al. (2016), a relationship exists between the sense of coherence, self‐regulated learning and academic performance in the subject of bioscience. In the first semester of the first year, most of the students are young and have no experience in patient care or higher education. This might be the most important factor inhibiting the learning process in bioscience regarding coherence. Nursing students might have different expectations related to their nurse education. Rognstad (2002) underlined that students’ expectations are related to human contact, to helping people in need and doing something useful. This indicates that the students may have other expectations than studying hard core subjects like biosciences in the first semester of their education.

As McVicar et al. (2015) claimed, some central factors were necessary in teaching bioscience, such as good learning environments and proper course organization. The learning environment and the course organization might be difficult to grasp in the first semester, with new learning platforms, and the high number of new students. In this project, the examination in biosciences is completed after only four months of teaching. The syllabus is comprehensive, and the students have learning activities in other subjects as well. Students must learn new concepts by heart, a study technic rarely used.

The flipped classroom as a method demands more resources than the auditorium lectures concerning the use of faculty. In our study, the flipped classroom included four teachers and one mentor, compared with the auditorium lectures approach with only one teacher. The flipped classroom requires more facilities, such as rooms and access to digital devices. One question might be whether this method is a waste of time and money when the students’ results are more or less the same as with traditional teaching methods. Bioscience is a subject that is essential to understanding the patients and their basic needs, their diseases and treatments, all aspects which constitute the foundation of nursing.

## 9 CONCLUSION

In this study, the results show there is no statistically significant difference between the groups, which indicate that the learning methods have equal effect on the examination results. The importance of the students’ entry grades in biosciences or natural sciences to nurse education deserves more attention and research.

A different research focus may also increase the understanding of how students learn biosciences, we suggest ethnographic studies, focusing on how the students study in these courses; furthermore, how much time they spend reading and what kind of devices work outside the classroom to enhance the students’ learning. Studies of teaching bioscience in nurse education suggest focusing more on relevance for the profession. If relevance is important, the students should be able study the subject throughout all three years of the programme.

### 9.1 Implications for education and further research

When implementing new learning methods, there is a need for a discussion on the resources used and the benefits of the methods. Huge expectations seem to be related to digitalizing educational activities in higher education and to swapping auditorium lectures to smaller seminar groups and individuals using digital devices. This study raises questions about this trend. Intervention studies in higher education might be challenging when students can choose to attend to the class or find other learning solutions. This factor makes educational interventions complex, creating interactive components within both groups.

We have studied grades as an outcome of the flipped classroom intervention. Further research is needed to explore flipped classroom and different learning outcomes, such as students’ activity level and their capability to use bioscience in nursing practice.

## CONFLICT OF INTEREST

No conflict of interest.

## ETHICAL APPROVAL

This project is approved by the Norwegian Data Protection Service (NSD), project number 614045. The students were informed of the research project before the bioscience courses, both written and oral presentations. The data used in this project is register‐data, no names or other recognizable data were used, so the consents were not required.

## Data Availability

Raw data were generated at Oslo Metropolitan University. Derived data supporting the findings of this study are available from the corresponding author [UK] on request.
